# Assessment of Knowledge and Attitudes Related to HIV/AIDS Among the Population With Increasing Incidence Rate

**DOI:** 10.7759/cureus.53451

**Published:** 2024-02-02

**Authors:** Nawaid H Khan, Mirza Masroor Beg, Mohammed Z Sarwar, Gulzat Z Kyzy, Tolkun Zhetkinbekova, Akyltai Mamatov, Aizirek Z Kyzy, Kudaibergen Osmonaliev, Mannap U Nurlanbek, Syed S Faisal, Hafiz Ahmad, Saleha Khanam, Shafee U Rehman

**Affiliations:** 1 Faculty of Medicine, Ala-Too International University, Bishkek, KGZ; 2 Department of Medical Microbiology and Immunology, RAK (Ras Al Khaimah) Medical and Health Sciences University, Ras Al Khaimah, ARE

**Keywords:** assessment, kyrgyzstan, attitude, knowledge, hiv/aids

## Abstract

Introduction

Lack of awareness and negative attitudes toward people living with HIV/AIDS (PLWHA) are key barriers to minimizing the transmission of HIV. Therefore, the present survey-based study aimed to assess the knowledge regarding HIV/AIDS and attitudes toward PLWHA.

Methods

In the present study, we collected data from 612 Kyrgyz national participants using a self-administered questionnaire.

Results

Among the participants, 59% (361) were females, and 41% (251) were males. The mean age of the participants was 26.23 (SD = 7.7) years. All participants were aware of HIV/AIDS, and 59.1% (362) agreed to have sufficient information about HIV/AIDS. Overall, the participants displayed a high level of knowledge about HIV/AIDS transmission, and 89.2% (546) of them were aware of sexual transmission of HIV/AIDS. Among the participants, 54% (330) believed that using condoms during sexual intercourse could prevent the transmission of HIV/AIDS. Concerning social attitudes, 17% (104) of the participants agreed that HIV-infected individuals should be isolated from society. Moreover, 39% (238) of them disagreed to work with PLWHA. The results of the study suggest that female participants were more aware of the modes of HIV/AIDS transmission than males. However, misconceptions regarding transmission routes were present in both genders.

Conclusion

The present study revealed that study participants had correct knowledge about HIV/AIDS transmission modes such as unsafe blood transfusion and injectable drug abuse. However, knowledge about unsafe tattooing and mother-to-baby mode of HIV/AIDS transmission was observed to be lower. Female participants were found to be more aware of HIV/AIDS transmission. There is a need to address the knowledge and awareness gap in the general population of Kyrgyzstan, especially among the male population.

## Introduction

The human immunodeficiency virus (HIV) continues to challenge public health over the world. Annual reports of the World Health Organization (WHO) on HIV/AIDS revealed that 650,000 HIV/AIDS-associated deaths occurred and 39.0 million people are living with HIV/AIDS globally, with 1.3 million new cases reported. It has been estimated that among HIV-infected persons, 85% of people knew their status and only 28.7 million (75%) are on antiretroviral therapy (ART) treatment, [1]. On the other hand, in Kyrgyzstan, according to the latest reports, approximately 10,000 people have been living with HIV/AIDS. The adult prevalence rate of HIV in Kyrgyzstan is 0.2 and the incidence per 1000 people is 0.19 [2].

However, in Kyrgyzstan, the first case of HIV was documented in 1987 in an immigrant, and after nine years, in 1996, the first case of HIV in a Kyrgyz citizen was documented [3].

An increased trend of new cases of HIV in Kyrgyzstan has been observed and the major route of transmission is the heterosexual mode [2]. However, the transmission of HIV can happen by contaminated blood and blood products, using infected needles, and surgical instruments; another important route of HIV spread is vertical transmission [4]. In the concerned population, initially before 2015, the major (>50%) mode of HIV transmission was injectable drug abuse and 36% was the sexual mode of transmission, but as per current data, the mode of transmission was shifted to sexual mode; 65% of HIV transmission is due to sexual mode while 26.9% were associated with abuse of injectable drugs [5]. The progression of HIV infection is mainly monitored by using patient HIV-1 plasma viral load (pVL) and CD4+ T-cell count [6]. The available antiretroviral drugs have greatly depreciated the mortality rate of HIV-infected individuals all over the world [7]. However, the major goals of ART are to maximize and durable suppression of viral load, restoration and preservation of immunologic function, improvement of quality of life, and reduction of HIV/AIDS-related morbidity and mortality [8]. The highly active antiretroviral therapy (HAART) is a combination of different classes of antiretroviral drugs, which effectively suppresses HIV replication, reduces disease progression, and minimizes the chance of developing drug resistance mutation [9]. The WHO has recommended a fixed single-dose combination of HAART that reduces the pill burden to achieve 95% drug adherence [10]. However, in the case of Kyrgyzstan, the 95-95-95, 2030 WHO global target regarding HIV/AIDS care and treatment is considerably behind. In 2021, 75% of people living with HIV/AIDS knew their HIV status, 50% of them were on ART, and only 45% had suppressed viral load [2]. Knowledge and awareness regarding HIV/AIDS are important strategies that could be used in the prevention and control of HIV/AIDS worldwide. The inappropriate knowledge, lack of awareness about HIV/AIDS, and negative attitude toward people living with HIV/AIDS (PLWHA) are key barriers to minimizing HIV transmission, treatment-associated positive outcomes, and also enhancing the HIV/AIDS-related stigma [11]. Unfortunately, there are limited data available from central Asia, including Kyrgyzstan, regarding HIV/AIDS knowledge and attitudes among the general population. The present survey-based study aimed to evaluate the knowledge regarding HIV/AIDS and attitudes toward people living with HIV/AIDS among Kyrgyz nationals.

## Materials and methods

Study design and population

A cross-sectional, face-to-face survey-based observational study using predesigned questionnaires was completed between May and August 2023 in Bishkek, Republic of Kyrgyzstan. In the present study, the participants were enrolled from different regions of Bishkek and the questionnaire was distributed among the people at popular spots, such as shopping malls, complexes, parks, squares, and universities, and responses were collected by investigators in English and Russian versions. The present study was ethically approved by the Ala-Too International University Ethics Board (AIUEB-M03-2023).

Enrolment of participants

A total of 612 participants were enrolled in the present study. Based on the inclusion criteria, all the participants were from Kyrgyzstan, and informed consent was taken before data collection and research objectives were fully explained to the participants with written informed consent. The non-Kyrgyzstan participants who were not willing to participate or refused to give consent were excluded from the study. The sample size was calculated by following the population size, confidence level (95%), and margin of error (5%). Participants were recruited from the Bishkek region of Kyrgyzstan, and face-to-face question's answers were recorded by visiting shopping malls, complexes parks, and universities. Incomplete information or recorded data were excluded from the study.

Data collection and questionnaire design

The questionnaire structure of the present study was adapted from previous similar relevant studies, which were conducted in different parts of the world with similar purposes [12-16]. The internal consistency of the questionnaire was calculated and observed Cronbach’s alpha coefficient was 0.76. For convenience and easy understanding of the survey, the English version of the questionnaire was translated into the Russian language. The initial part of the questionnaire concerned with participants’ socio-demographic characteristics was assigned to assess the knowledge, and awareness attitude about HIV/AIDS in Kyrgyzstan. For the socio-demographic data, there were six question items and the source of knowledge was recorded. The majority of the 16 questions on HIV/AIDS dealt with broad concepts like the virus's transmission mode and common misunderstandings about the disease. Respondents could choose between yes/no and "don't know" as their choices to record the responses. Furthermore, the attitude regarding the use of condoms to prevent HIV transmission (1 = strongly disagree; 2 = disagree; 3 = neither agree nor disagree; 4 = agree; 5 = strongly agree) and two questions about attitudes toward people living with HIV/AIDS were included.

Statistical analysis

All the data were recorded in Microsoft Excel (Microsoft Corporation, Redmond, WA). Statistical analysis was performed using SPSS version 21.0 (IBM Corp., Armonk, NY). The mean and SD were calculated for all the study variables. A p-value of <0.05 was considered significant in all the statistical analyses. The data were presented in tables and p-values among the variable groups were calculated by the chi-square test, and graphical representations have been used according to the distribution of data.

## Results

Demographic characteristics of the participants

A total of 612 participants' data were collected and depicted in Table 1. Among all participants, 59% (362) were females, and 41% (251) were males. A majority (64.2%, 393) of participants were in the age group of 18 to <25 years and the mean age was (26.23, SD = 7. 7). The education level of the participants revealed 69.9% (426) were graduates, 27.1% (166) were in secondary schooling, 2.6% (16) were in primary schooling, and 0.7% (4) were uneducated.

**Table 1:** General and demographic profile of the study participants.

Variables	N (%)
Gender	
Male	251 (41%)
Female	361 (59%)
Age (years)	
18 to ≤25	393 (64.22%)
26 to ≤35	100 (16.34%)
36 to ≤45	91 (14.87%)
>45	28 (4.57%)
Mean, SD	26.23, SD = 7.7
Education level	
College & above	426 (69.6%)
Secondary schooling	166 (27.1%)
Primary schooling	16 (2.6%)
Illiterate	4 (0.7%)
Employment status	
Unemployed	261 (42.6%)
Daily wage employed	90 (14.8%)
Regular employed	261 (42.6%)

Source of information on HIV/AIDS among participants

**Figure 1:**
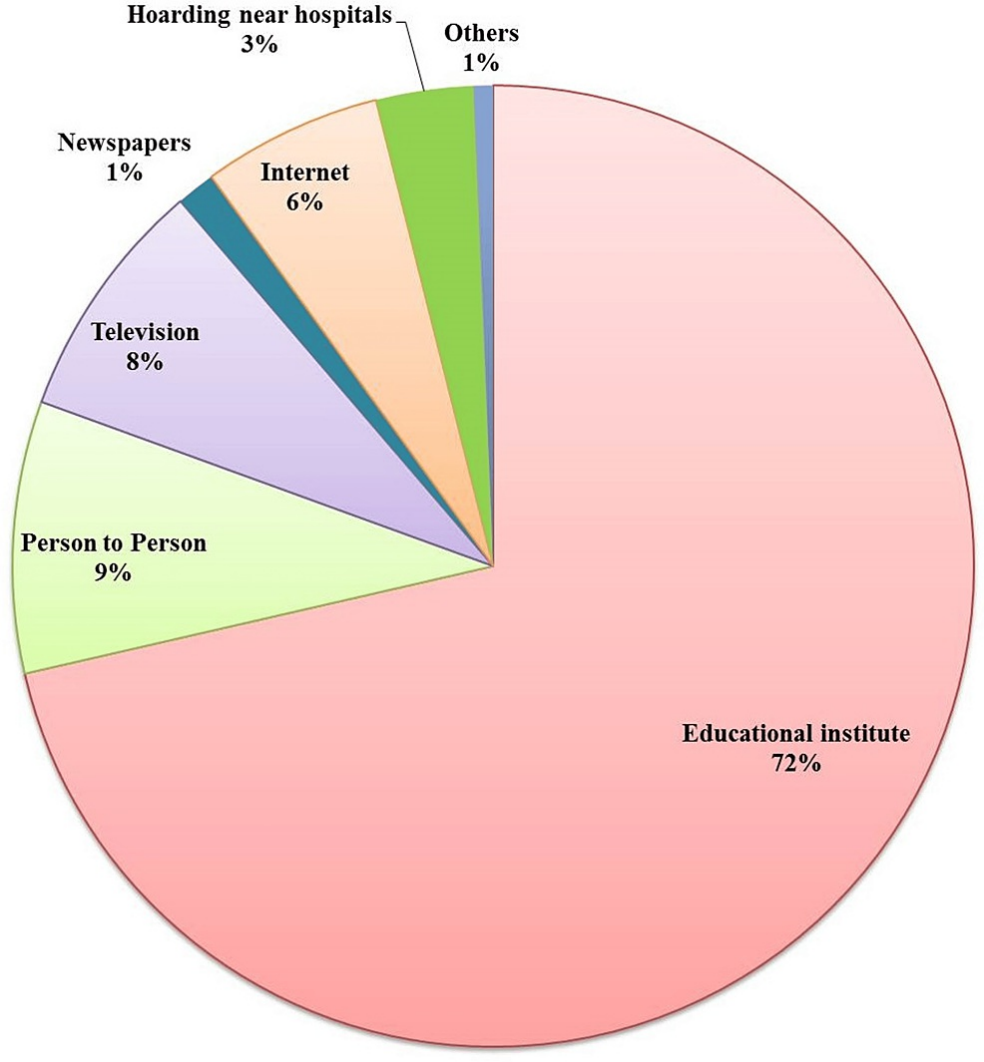
Source of information about HIV/AIDS among the participants.

Participants were asked for sources of information on HIV/AIDS and it was observed that 72% (440) of participants had information from educational institutes, 9% (55) from person to person, 8% (48) from television, 6% (36) from internet, 3% (18) from hoarding at hospitals, and 1% (6) of participants had information from newspapers and others, respectively (Figure 1).

General information and knowledge of participants about HIV/AIDS transmission

We observed that all participants had heard about HIV/AIDS. Among them, 59.1% (362) of participants agreed that they had sufficient information about HIV/AIDS. The following statistics are presented for the participants: 69.6% (426) knew what causes AIDS, 44.3% (271) thought that HIV and AIDS are the same, 55.7% (341) wanted a cure, 27.8% (170) thought that HIV is a curable disease, and 30% (183) thought that there is no vaccine for HIV. Additionally, 70% (429) had poor knowledge about the vaccine. However, 30% (183) of the participants responded there is no vaccine for HIV, and 70% (429) of the participants had poor knowledge about the HIV vaccine. It was observed that 38% (233) of participants had information about the availability of treatment while 62% (379) did not have information about the treatment option. We also accessed the participants' knowledge regarding the mode of HIV transmission. Of the participants, 89.2% (546) were aware that HIV/AIDS is a sexually transmitted disease. Most of the participants (92.8%, 568) knew that unsafe blood transfusion can transmit HIV and 91.2% (558) of participants responded that sharing needles or syringes can transmit HIV during illegal drug abuse. Tattooing as a source of HIV transmission was responded to by 67% (410), as well as 70.6% (432) responded that the HIV-infected mother can transmit the virus to the baby. The misconception regarding HIV/AIDS transmission in the study population was that 25.5% (156) of the participants believed that sharing a toilet seat can transmit HIV, 23.2% (142) believed coughing and sneezing could transmit the disease, 31.1% (190) believed that sharing a glass of water can transmit HIV, and 37.6% (230) believed that mosquito bites could lead to HIV/AIDS (Table 2).

**Table 2:** Knowledge regarding HIV/AIDS (n = 612).

Question items for general information about HIV/AIDS (n = 7)	Responses (n = 612)	
	Yes, n (%)	No, n (%)
Did you ever hear about HIV/AIDS?	612 (100%)	-
Do you think you have sufficient information about HIV/AIDS?	362 (59.1%)	250 (40.9%)
AIDS is caused by a virus.	426 (69.6%)	186 (30.4%)
HIV and AIDS are the same thing.	271 (44.3%)	341 (55.7%)
There is a cure for AIDS.	170 (27.8%)	442 (72.2%)
There is no vaccine available for HIV.	429 (70%)	183 (30%)
There are some drugs available for the treatment of AIDS.	233 (38%)	379 (62%)
Question items for knowledge regarding the transmission mode of HIV/AIDS (n = 9)		
HIV/AIDS is a sexually transmitted disease.	546 (89.2%)	66 (10.8%)
A person can get HIV from sharing a toilet seat.	156 (25.5%)	456 (74.5%)
Coughing and sneezing can spread HIV.	142 (23.2%)	470 (76.8%)
A mosquito bite can spread HIV.	230 (37.6%)	382 (62.4%)
A person can get HIV by sharing a glass of water with someone who has HIV.	190 (31.1%)	422 (68.9%)
It is possible to get HIV when a person gets a tattoo.	410 (67%)	202 (33%)
HIV can be transmitted by unsafe blood transfusion.	568 (92.8%)	44 (7.2%)
It is possible to get HIV when a person shares needles and syringes while taking illegal drugs or drug addiction.	558 (91.2%)	54 (8.8%)
A pregnant woman with HIV can give the virus to her baby.	432 (70.6%)	180 (29.4%)

Attitude toward condom use and PLWHA among participants

**Figure 2:**
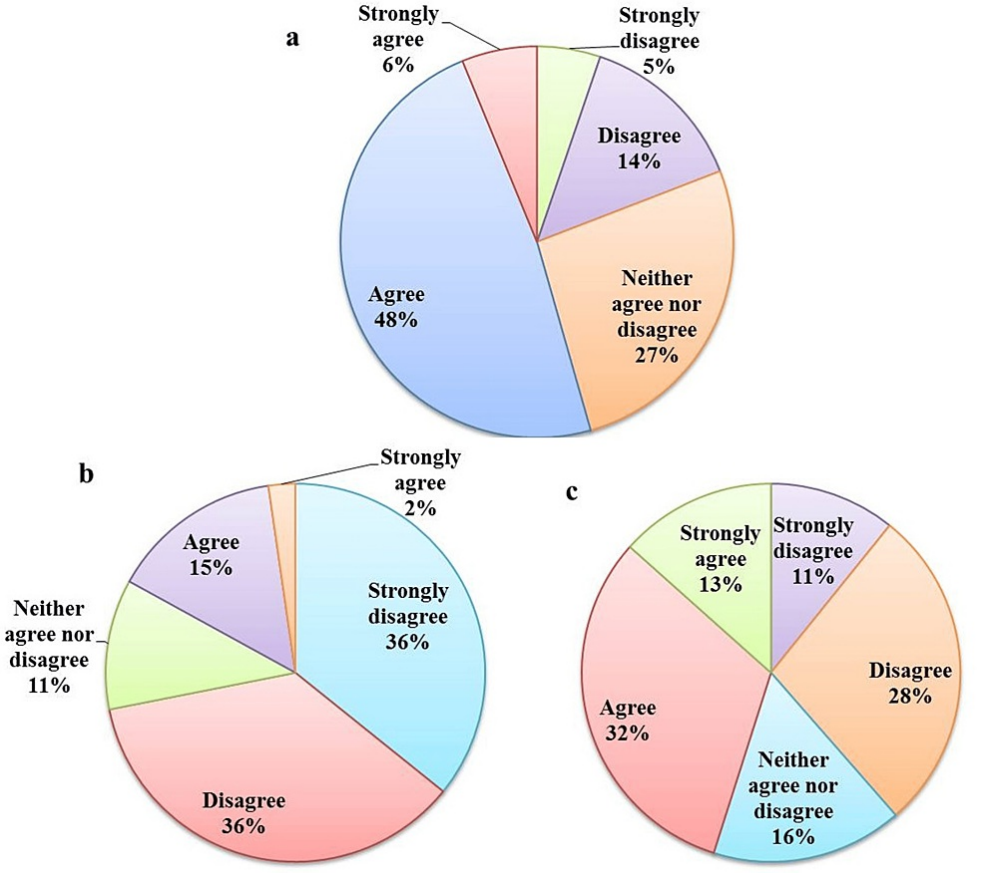
Demonstration of attitude toward condom use and stigma among participants. (A) The use of condoms can prevent HIV transmission. (B) People with HIV/AIDS should be separated from society. (C) Do you agree to work with HIV/AIDS-positive individuals?

The five-point Likert scale questions were used to evaluate the attitude toward condoms and PLWHI (Figure 2). Approximately 6% (36) of participants strongly agreed that HIV transmission can be prevented by using a condom during sexual intercourse while 48% (293) of participants agreed that HIV transmission can be prevented by using a condom during sexual intercourse. However, 27% (165) neither agreed nor disagreed that HIV transmission can be prevented by using a condom during sexual intercourse. It was also observed that 14% (85) of participants disagreed that HIV transmission could be prevented by using a condom during sexual intercourse and 5% (30) strongly disagreed (Figure 2A). We collected the data regarding the social existence of HIV patients in society and observed that 36% (220) of participants strongly disagreed with not separating HIV patients from society and only 2% (12) strongly agreed to be isolated from society while 36% (220) again disagreed not to isolate the HIV patients from society. Still, 15% (91) of participants think that HIV patients should be isolated from society and 11% (67) did not show any agreement or disagreement (Figure 2B). We also collected data regarding the work culture with HIV patients and observed that regarding willingness to work with HIV patients, 13% (79) strongly agreed, 32% (195) agreed, 11% (67) strongly disagreed, 36% (220) disagreed, and 16% (97) neither agreed nor disagreed (Figure 2C).

Gender-based knowledge comparison of HIV/AIDS transmission

We compared the knowledge of HIV/AIDS transmission and related misconceptions among male and female participants (Table 3). Male and female participants had poor information about the transmission of HIV by sharing a toilet seat. It was observed that 30.2% (76) of males said sharing a toilet could spread HIV while 22.1% (80) of females responded the same (p = 0.02). We observed that 35.9% (90) of male participants responded that sharing a glass of water could transmit HIV while the same was recorded by 27.7% (100) of female participants (p = 0.03). Misconception about HIV transmission by coughing and sneezing was evaluated and it was observed that 25.5% (64) of males and 21.6% (78) of females responded yes, while 40.2% (101) of males and 35.7% (129) of females had misconceptions about the mode of transmission of HIV by mosquito bites; it was an interesting finding of the study. Most of the participants (88.1% (221) males and 90% (325) females) were aware of the sexual transmission of HIV/AIDS. It was observed that tattooing transmits HIV in humans, and in our research survey, we observed that 61% (153) of males and 71.1% (257) of females were aware of tattooing practice as the mode of HIV transmission (p = 0.008). Of the participants, 96% (241) of males and 90.6% (327) of females were aware of the transmission of HIV by blood transfusion (p = 0.01), 89.6% (225) of males and 92.2% (333) of females were aware that HIV transmission can happen through sharing of needle and syringe, and 65.7% (165) males and 74% (267) females knew about HIV transmission through HIV-infected mother to babies (p = 0.02) (Table 3).

**Table 3:** Gender-based comparisons of the knowledge about HIV/AIDS transmission.

Knowledge about HIV/AIDS transmission	Male (n = 251)		Female (n = 361)		P-value	
	Yes, n (%)	No, n (%)	Yes, n (%)	No, n (%)		
HIV/AIDS is a sexually transmitted disease	221 (88.1%)	30 (11.9%)	325 (90%)	36 (10%)	0.43	
A person can get HIV from sharing a toilet seat	76 (30.2%)	175 (69.8%)	80 (22.1%)	281 (77.9%)	0.023	
Coughing and sneezing can spread HIV	64 (25.5%)	187 (74.5%)	78 (21.6%)	283 (78.4%)	0.26	
Mosquito bite can spread HIV	101 (40.2%)	150 (59.8%)	129 (35.7%)	232 (64.3%)	0.25	
A person can get HIV by sharing a glass of water with someone who has HIV	90 (35.9%)	161 (64.1%)	100 (27.7%)	261 (72.3%)	0.032	
It is possible to get HIV when a person gets a tattoo	153 (61%)	98 (39%)	257 (71.1%)	104 (28.9%)	0.008	
HIV can be transmitted by unsafe blood transfusion	241 (96%)	10 (4%)	327 (90.6%)	34 (9.4%)	0.010	
It is possible to get HIV when a person shares a needle and syringe while taking illegal drugs or drug addiction	225 (89.6%)	26 (10.4%)	333 (92.2%)	28 (7.8%)	0.26	
A pregnant woman with HIV can give the virus to her baby	165 (65.7%)	86 (34.3%)	267 (74%)	94 (26%)	0.028	

## Discussion

The knowledge and awareness regarding HIV/AIDS in the general population is a major preventive tool for reducing the spread of the HIV pandemic. The data published by the WHO presented a reduction in new HIV cases due to educational and awareness activities. Proper implementation of preventive measures is the most effective strategy for HIV/AIDS testing, treatment, and monitoring [1]. Unfortunately, in Kyrgyzstan, current data present a remarkable increase in new cases of HIV [2]. This is, to our knowledge, the first survey-based study in Kyrgyzstan performed to assess the level of knowledge and attitude related to HIV/AIDS among the Kyrgyz population. We believe that our findings may contribute positively to policymakers and encourage them to make more effective educational and awareness activities for the prevention of HIV spread. The majority of participants in the current survey were sexually active (average age = 26.23 years) and this awareness may contribute to the decline in the incidence of new HIV cases by adopting preventive measures such as using condoms, safe practice of tattooing, and avoiding drug abuse. All the participants had heard about HIV/AIDS at the time of the survey; however, approximately 40.9% (250) of them had a lack of information regarding HIV/AIDS.

A similar study in Kazakhstan performed by Sandgren et al. in 2008 reported that 96% of participants heard about HIV/AIDS [12]. A recent study by Qashqari et al. in 2022 reported that among the general population, approximately 91% of participants heard about HIV/AIDS [13]. Educational institutions play an important role in the awareness of HIV/AIDS-related knowledge among the population across countries. In Kyrgyzstan, we observed that 71.4% (437) of participants received information from educational institutes and only 18.8% (115) through media sources such as television, the internet, newspapers, and hoardings near hospitals. A similar finding was reported by Thanduxolo et al. in 2021, where the majority of participants received first-time information about HIV/AIDS from their teachers [14]. In the current scenario worldwide, people are getting health-related knowledge from electronic media and the internet. However, studies from different countries such as Kazakhstan, Sudan, Pakistan, and Turkey reported that media sources, particularly electronic media, are major sources of spreading information about HIV/AIDS awareness [12,15-17].

The overall level of general information regarding HIV/AIDS among the study population varies differently with specific question items. Among the participants, 69.6% (425) knew about the causative agents of AIDS and 72.2% (442) knew it was not a curable disease. However, for the vaccine and antiretroviral drugs, the level of information observed was poor, as more than 50% of participants had no information about the differences between HIV and AIDS, and also for the availability of effective vaccines against HIV/AIDS and antiretroviral drug for treatment. The literacy rate of participants was very high (98.3%, 602) while the level of general information about HIV/AIDS was poor. A study by Dzah et al. (2019) reported that 59.2% of the youth population had information that HIV is a curable disease [18]. In contrast to our finding, Alwafi et al. in 2019 reported that 57.9% of participants from Saudi Arabia had correct information about the availability of effective treatment of HIV/AIDS [19]. For the remaining information, a previous study by Ayranci (2005) observed similar findings [17]. It is a well-established fact that HIV/AIDS is majorly transmitted via the sexual mode of transmission, particularly the heterosexual mode played a major role in the global spread of HIV/AIDS. As per the available data in the study population, the major mode of transmission is sexual mode and the second most common mode is injectable drug abuse [2]. However, in our study, we observed that overall, 89.2% (546) of participants knew that sexual intercourse is the major mode of HIV/AIDS transmission and the remaining 10.8% (66) of participants did not know about this. Our results demonstrated that 92.8% (568) of participants had appropriate knowledge regarding the spread of HIV/AIDS through unsafe blood transfusion practices, which was highly encouraging. Our participants had sound knowledge about the injectable drug abuse mode of HIV/AIDS transmission. Our study suggested that participants have a good level of knowledge about the mode of HIV/AIDS transmission, and many studies favor our present findings [13,14,18-20]. Furthermore, knowledge among the participants about HIV/AIDS transmission by tattooing and mother-to-child transmission is less compared to other modes of transmission. However, our result shows that the participants knew about the mode of HIV/AIDS transmission, but it was also observed that there were many misconceptions regarding the mode of transmission among participants such as coughing and sneezing, sharing toilet seats, sharing utensils with HIV-infected individuals, and mosquito bite can transmit HIV/AIDS, identifying the gap analysis and importance of this survey in this population. Proper knowledge of the different modes of HIV/AIDS transmission in the general population is one of the important factors for the prevention of HIV/AIDS [18-21].

In the study population, the attitude regarding the use of condoms as a major prevention tool of HIV/AIDS transmission showed positive knowledge and attitude toward the use of condoms to prevent HIV/AIDS transmission. In contrast to our finding, in a recent study from Iran, Shokoohi et al. (2016) reported that 30% of the adult population had a negative attitude toward using condoms for the prevention of HIV/AIDS [22]. The use of condoms during sexual intercourse has an important role in reducing individual risk of HIV transmission, which may result in decreasing new cases of HIV/AIDS in the population. Thus, spreading awareness about the importance of condom use among sexually active age groups definitely may decrease the incidence of HIV/AIDS at the national level as well as globally [13,16,20,22].

In this study, the participant's attitudes toward PLWHA were diverse. Of the participants, 17% (104) believed that HIV/AIDS-infected individuals should be isolated from society and 39% (239) were not willing to share the workplace with infected individuals, indicating the prevailing social stigma associated with HIV in the current study. Study participants showed positive attitudes toward HIV/AIDS-infected patients for social acceptance. In contrast to our findings, studies from the Middle East and North Africa (MENA) region reported that 40.73% and 60.1% of participants from Saudi Arabia and Bahrain, respectively, believed HIV/AIDS-infected patients should be isolated from society. These disagreements may be associated with the cultural context of the study population [19,23]. The negative attitudes toward PLWHA are attributed to stigmatization; however, the social stigma is a major barrier to positive outcomes of HIV/AIDS-related preventive measurement and treatments. On the other hand, by spreading correct knowledge and awareness among the community, the HIV/AIDS-associated social stigma can be minimized. Therefore, in recent years, with the help of campaigns, advertisements, social media, and the internet, the knowledge and awareness of HIV/AIDS increased globally [13,14,18-20]. So, there is a need, particularly in middle- and low-income countries, to decrease social stigma toward HIV/AIDS, ensuring social protection and acceptance. Along with this, improving the knowledge and positive attitudes among the general population toward PLWHA have been considered critical factors for the handling of the epidemic [16,18,20,21]. Notably, our results showed that the female participants had an overall high level of knowledge regarding the common mode of HIV/AIDS transmission as compared to the male participants. In contrast to our findings, the studies from Uganda and South Africa reported that knowledge about HIV transmission is high among males as compared to female participants [24,25]. Furthermore, for other possible modes of HIV/AIDS transmission, for example, unsafe tattoos (28.9%, 104), unsafe blood transfusion practice (9.4%, 34), mother-to-baby transmission (26%, 94), and injectable drug abuse (7.8%, 28), female participants believed that HIV/AIDS cannot be transmitted via these modes. However, misconceptions regarding HIV/AIDS transmission were also observed among female participants, where some of them believed that transmission was possible by sharing of toilet seat (22.1%, 80), coughing and sneezing (21.6%, 78), sharing a glass of water (27.7%, 100), and mosquito bite (35.7%, 129). Among these misconceptions, transmission via mosquito bite was more common in the study population, including male participants (40.2%, 101). Such misconceptions were not unique and previous studies reported even higher levels of similar misconceptions among their studies population [26,27]. In contrast to our findings, a recent study from Bangladesh observed a high level of misconceptions about HIV/AIDS transmission among female participants [2]. The difference in results may be due to the level of education (99.3%, 608) of study participants. The present study was limited to urban areas, which could have differences in observation compared to rural area participants.

## Conclusions

The study revealed that the majority of participants were aware of the routes of HIV/AIDS transmission, such as sexual mode, unsafe blood transfusion, and injectable drug abuse. Knowledge of other possible routes of HIV/AIDS transmission, such as unsafe tattooing and mother-to-baby transmission, was marginally low. Furthermore, the majority of participants showed a positive attitude toward PLWHA but the attitude regarding the use of condoms for HIV/AIDS prevention was not satisfactory. Misconceptions of HIV/AIDS transmission, such as sharing toilet seats, coughing and sneezing, sharing a glass of water, and mosquito bites, were noted in participants. Participants had misinformation about the availability of an effective vaccine against HIV/AIDS and drugs, and some believed HIV/AIDS is a curable disease due to poor awareness. Hence, educational campaigns and awareness programs in schools, colleges, and universities could be more helpful in raising awareness about HIV/AIDS and reducing stigma toward PLWHA. The study suggested that promoting awareness through electronic media and social media platforms could be a good tool to target the potential population.

## References

[1] (2023). World Health Organization. HIV and AIDS. https://www.who.int/news-room/fact-sheets/detail/hiv-aids/.

[2] Bhowmik J, Biswas RK (2022). Knowledge about HIV/AIDS and its transmission and misconception among women in Bangladesh. Int J Health Policy Manag.

[3] Sivay MV, Totmenin AV, Zyryanova DP (2021). Characterization of HIV-1 epidemic in Kyrgyzstan. Front Microbiol.

[4] Santoro MM, Perno CF (2013). HIV-1 genetic variability and clinical implications. ISRN Microbiol.

[5] Zhao F, Benedikt C, Wilson D (2020). Tackling the world’s fastest-growing HIV epidemic: more efficient HIV responses in Eastern Europe and Central Asia. Human Development Perspectives.

[6] Mellors JW, Muñoz A, Giorgi JV (1997). Plasma viral load and CD4+ lymphocytes as prognostic markers of HIV-1 infection. Ann Intern Med.

[7] Tseng A, Seet J, Phillips EJ (2015). The evolution of three decades of antiretroviral therapy: challenges, triumphs and the promise of the future. Br J Clin Pharmacol.

[8] Günthard HF, Saag MS, Benson CA (2016). Antiretroviral drugs for treatment and prevention of HIV infection in adults: 2016 recommendations of the International Antiviral Society-USA Panel. JAMA.

[9] Cohan D, Feakins C, Wara D (2005). Perinatal transmission of multidrug-resistant HIV-1 despite viral suppression on an enfuvirtide-based treatment regimen. AIDS.

[10] Iacob SA, Iacob DG, Jugulete G (2017). Improving the adherence to antiretroviral therapy, a difficult but essential task for a successful HIV treatment-clinical points of view and practical considerations. Front Pharmacol.

[11] Herek GM, Capitanio JP, Widaman KF (2002). HIV-related stigma and knowledge in the United States: prevalence and trends, 1991-1999. Am J Public Health.

[12] Sandgren E, Sandgren S, Urazalin M, Andersson R (2008). HIV/AIDS awareness and risk behaviour among pregnant women in Semey, Kazakhstan, 2007. BMC Public Health.

[13] Qashqari FS, Alsafi RT, Kabrah SM, AlGary RA, Naeem SA, Alsulami MS, Makhdoom H (2022). Knowledge of HIV/AIDS transmission modes and attitudes toward HIV/AIDS infected people and the level of HIV/AIDS awareness among the general population in the Kingdom of Saudi Arabia: a cross-sectional study. Front Public Health.

[14] Thanduxolo F (2021). Knowledge, attitude and practices regarding HIV and aids among high school learners in South Africa. Open AIDS J.

[15] Nasir EF, Astrøm AN, David J, Ali RW (2008). HIV and AIDS related knowledge, sources of information, and reported need for further education among dental students in Sudan--a cross sectional study. BMC Public Health.

[16] Zafar M, Nisar N, Kadir M, Fatmi Z, Ahmed Z, Shafique K (2014). Knowledge, attitude and practices regarding HIV/AIDS among adult fishermen in coastal areas of Karachi. BMC Public Health.

[17] Ayranci U (2005). AIDS knowledge and attitudes in a Turkish population: an epidemiological study. BMC Public Health.

[18] Dzah SM, Tarkang EE, Lutala PM (2019). Knowledge, attitudes and practices regarding HIV/AIDS among senior high school students in Sekondi-Takoradi metropolis, Ghana. Afr J Prim Health Care Fam Med.

[19] Alwafi HA, Meer AMT, Shabkah A, Mehdawi FS, El-Haddad H, Bahabri N, Almoallim H (2018). Knowledge and attitudes toward HIV/AIDS among the general population of Jeddah, Saudi Arabia. J Infect Public Health.

[20] John NN, Krishnan AK, Doddayya H (2021). A study on knowledge attitude and practices regarding HIV/AIDS among general population in a community of Kottarakkara, Kerala. Int J Commun Med.

[21] Hong SY, Thompson D, Wanke C (2012). Knowledge of HIV transmission and associated factors among HIV-positive and HIV-negative patients in rural Kenya. J AIDS Clin Res.

[22] Shokoohi M, Karamouzian M, Mirzazadeh A, Haghdoost A, Rafierad AA, Sedaghat A, Sharifi H (2016). HIV knowledge, attitudes, and practices of young people in Iran: findings of a national population-based survey in 2013. PLoS One.

[23] Janahi EM, Mustafa S, Alsari S, Al-Mannai M, Farhat GN (2016). Public knowledge, perceptions, and attitudes towards HIV/AIDS in Bahrain: a cross-sectional study. J Infect Dev Ctries.

[24] Nabunya P, Byansi W, Muwanga J (2021). Gender, HIV knowledge and prevention attitudes among adolescents living with HIV participating in an economic empowerment intervention in Uganda. AIDS Care.

[25] Rohleder P, Eide AH, Swartz L, Ranchod C, Schneider M, Schür C (2012). Gender differences in HIV knowledge and unsafe sexual behaviours among disabled people in South Africa. Disabil Rehabil.

[26] Yoo H, Lee SH, Kwon BE, Chung S, Kim S (2005). HIV/AIDS knowledge, attitudes, related behaviors, and sources of information among Korean adolescents. J Sch Health.

[27] Choy KK, Huo ALK, Lee JER, Sabapathy MG, Jing OJ, Jutti RC (2013). Frequent misconceptions and low-to-moderate knowledge of HIV and AIDS amongst high-school students in Malaysia. Int Sch Res Notices.

